# Inertial Measurement Unit Based Upper Extremity Motion Characterization for Action Research Arm Test and Activities of Daily Living

**DOI:** 10.3390/s19081782

**Published:** 2019-04-14

**Authors:** Hyung Seok Nam, Woo Hyung Lee, Han Gil Seo, Yoon Jae Kim, Moon Suk Bang, Sungwan Kim

**Affiliations:** 1 Department of Biomedical Engineering, Seoul National University College of Medicine, Seoul 03080, Korea; ignite31@snu.ac.kr (H.S.N.); v3night9@snu.ac.kr (W.H.L.); 2 Department of Rehabilitation Medicine, Seoul National University Hospital, Seoul 03080, Korea; hangilseo@snu.ac.kr; 3 Interdisciplinary Program for Bioengineering, Seoul National University Graduate School, Seoul 08826, Korea; kyj182731@naver.com; 4 Department of Rehabilitation Medicine, Seoul National University College of Medicine, Seoul 03080, Korea; 5 Institute of Medical and Biological Engineering, Seoul National University, Seoul 03080, Korea

**Keywords:** inertial measurement unit, upper extremity, motion, action research arm test, activities of daily living

## Abstract

In practical rehabilitation robot development, it is imperative to pre-specify the critical workspace to prevent redundant structure. This study aimed to characterize the upper extremity motion during essential activities in daily living. An IMU-based wearable motion capture system was used to access arm movements. Ten healthy subjects performed the Action Research Arm Test (ARAT) and six pre-selected essential daily activities. The Euler angles of the major joints, and acceleration from wrist and hand sensors were acquired and analyzed. The size of the workspace for the ARAT was 0.53 (left-right) × 0.92 (front-back) × 0.89 (up-down) m for the dominant hand. For the daily activities, the workspace size was 0.71 × 0.70 × 0.86 m for the dominant hand, significantly larger than the non-dominant hand (*p* ≤ 0.011). The average range of motion (RoM) during ARAT was 109.15 ± 18.82° for elbow flexion/extension, 105.23 ± 5.38° for forearm supination/pronation, 91.99 ± 0.98° for shoulder internal/external rotation, and 82.90 ± 22.52° for wrist dorsiflexion/volarflexion, whereas the corresponding range for daily activities were 120.61 ± 23.64°, 128.09 ± 22.04°, 111.56 ± 31.88°, and 113.70 ± 18.26°. The shoulder joint was more abducted and extended during pinching compared to grasping posture (*p* < 0.001). Reaching from a grasping posture required approximately 70° elbow extension and 36° forearm supination from the initial position. The study results provide an important database for the workspace and RoM for essential arm movements.

## 1. Introduction

In the last decade, there have been dramatic improvements in rehabilitation robots and kinematic analyses of the upper extremities. Many types of multi-axis exoskeletons have been developed, as well as relatively simple end-effector type robots [1,2,3,4,5]. Exoskeletons are commonly defined as having a structure in which the robot joints correspond to human joints, whereas end-effector type robot structures do not correspond to human anatomical structures [1,6]. However, even in exoskeletons, the angular movements of human and robot joints do not exactly match. This discrepancy comes from the fundamental difference in that exoskeleton joints have mechanical joint axes with their corresponding motors, whereas human joints consist of bones, muscle and tendons, and soft tissues [7]. Therefore, the goal of exoskeleton rehabilitation robot development should not focus on perfectly resembling the human arm joint and structure, but rather on designing a modified structure based on a better understanding of human kinematics.

Many types of sensors are used for a motion analysis of the upper extremities including electromagnetic sensors, mechanical sensors, optical sensors, and inertial sensors. Aizawa et al. [8] reported the range of motions (RoM) of the major upper extremity joints during selected activities of daily living (ADL), using a commercial electromagnetic sensor system. Gates et al. [9] also quantified the RoM of the upper extremities during eight ADLs using reflective sensors. Kim et al. [10] conducted a kinematic analysis of drinking movements using reflective markers. Chen and Lum [11] used a spring-operated exoskeleton to compare the RoM with and without robotic assistance during the given tasks, where the angles were evaluated using mechanical sensors within the robot. Perez et al. [12] introduced a portable motion analysis for the upper limbs using inertial measurement unit (IMU) sensors. A recent review showed that accelerometers and IMUs are most frequently used devices for an analysis of upper limb motion [13]. Although many of these reports present quantitative angular values during specific ADL tasks, assessments focusing on clinically relevant applications of the motion data remain scarce. 

To minimize the size and complexity of neurorehabilitation robots, the number of axes and the workspace of a robotic hand or end-effector should be minimized; at the same time, however, essential tasks need to be performable during daily activities. From the viewpoint of performing a specific task, although the human performance using an arm may seem similar to the actuation of a robot, when considering the mechanism of the performance, they are significantly different. Moreover, it is possible to state that biological and engineering mechanisms are in significant opposition [7]. It is important to have a database on the position and joint angles while performing essential daily activities; however, the movement patterns in healthy subjects and stroke patients differ significantly, and the exoskeleton cannot be actuated in exactly the same manner as a human limb. It is necessary to create a design that patients may not only wear but also actuate in an appropriate manner to help the movement of a paralyzed limb and induce neuroplasticity.

The purpose of this study was to provide a database on the dimensions of the essential workspace and the RoM of the major upper extremity joints during the normal motion of healthy subjects from clinical and practical perspectives.

## 2. Materials and Methods

In this section, we present the IMU-based motion capture system used in this study with validation protocol, followed by participants information and task protocols. Detailed information on extracted parameters and statistical methods are also provided. 

### 2.1. Upper Extremity Motion Capture System and Its Validation

**Figure 1:**
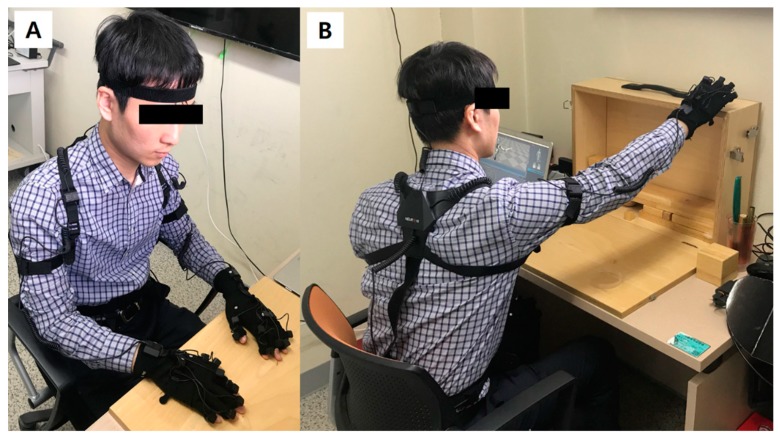
(**A**) A volunteer subject is wearing the IMU-based upper extremity motion capture system. (**B**) The subject is performing a task in the Action Research Arm Test.

For motion capture of the upper extremities, Perception Neuron^®^ (Noitom Ltd., Beijing, China), a wearable multi-IMU based modular motion capture system was used. In this study, we utilized 25 IMU sensors for the upper body assessment; three sensors for the body axis, four sensors for each arm, and seven sensors for each hand including the fingers (Figure 1). User-interface software, Axis Neuron (Noitom Ltd., Beijing, China), was applied for motion recording and data extraction. The sampling rate of the data was set to 60 Hz.

To validate the accuracy and consistency of the system, root mean square error (RMSE) analyses for the elbow flexion/extension and wrist dorsiflexion/volarflexion axes were performed using an electro-goniometer as a reference. During real motion with the system worn on the body, it is not possible to isolate single joint movements in a single plane with all other joint being fixed. Therefore, coefficient of variation (CoV) analyses for forearm supination/pronation and elbow flexion/extension for the angles from a gyrosensor, and the z-axis (up-down) and y-axis (front-back) distances from accelerometers in the forearm and hand sensors were conducted using the data collected during the tasks.

### 2.2. Participants

Ten healthy volunteers (six males, four females) were recruited for this study, and participated after providing written informed consent. Their mean age was 29.3 ± 4.7 years (age range: 23–35). 

### 2.3. Tasks and Procedure

All subjects wore the IMU sensor based motion capture system on both upper extremities. After sensor calibration, they performed all 19 test items of the Action Research Arm Test (ARAT) with using both their right and left hands alternatively [14]. ARAT consists of four domains: domain 1 includes six grasp and reaching tasks with various size of wooden blocks, ball, or a stone; domain 2 consists of four grip activities such as pouring water from glass to glass or putting a hollow tube through a stick; domain 3 includes six pinch and reaching tasks using various size of marbles using different fingers; and domain 4 consists of four gross movements placing the hand on three different parts of the head [14]. They also performed six pre-specified ADL tasks: (1) opening a water bottle and drinking, (2) peeling off a banana, (3) buttoning and unbuttoning a shirt, (4) combing their hair, (5) squeezing toothpaste from a tube and brushing their teeth, and (6) turning a door knob. These pre-specified ADL tasks were selected from the survey results from our previous study which evaluated the practical needs of stroke patients owing to their hemiplegia [15]. During the ADL tasks, the subjects were instructed to perform the task in the most natural way possible, without specifying which hand to use to hold or manipulate the object.

### 2.4. Extracted Parameters

Using Axis Neuron (Noitom Ltd., Beijing, China) software, acceleration and position data of the wrist and hand sensors from the accelerometer, and the Euler angles for the sensors of all major joints with reference to their proximal segment sensors during the ARAT and ADL tasks, were extracted. For each ARAT domain and ADL task, the size of the workspace in three orthogonal coordinates and the angular position and RoM for each upper extremity joint were calculated. For a sub-analysis, grasping/pinching and reaching movements when conducting the tasks in ARAT domains 1 and 3 were additionally analyzed regarding the initial grasping/pinching position and RoM during a reaching movement. 

### 2.5. Statistical Analysis

For validation purposes, the intra-subject covariance and inter-subject covariance were both calculated for repetitive grasping/pinching and reaching tasks. Paired *t* tests were conducted to compare the workspace dimensions and RoM between dominant and non-dominant arms. Paired *t* tests were also conducted for a comparison of the major joint angles in the grasping/pinching position and reaching position, the initial position between grasping and pinching, and the reaching position from grasping and pinching. The statistical program SPSS ver. 25 (SPSS Inc., Chicago, IL, USA) was used for analysis. A *p* value of less than 0.05 was considered statistically significant.

## 3. Results

The validation results followed by data analysis on movement characteristics are provided in this section.

### 3.1. Validation of Upper Extremity Motion Capture System

The range of RMSE for the elbow flexion/extension angle ranged from 2.11° to 4.75° (3.61 ± 1.32°), and 0.42° to 1.22° (0.85 ± 0.40°) for wrist dorsiflexion/volarflexion angle. During the reaching task, the mean change in forearm supination/pronation was 36.65 ± 6.98°, with an intra-subject CoV of 17.29% and inter-subject CoV of 19.05%. The change in elbow flexion/extension was 69.96 ± 16.89°, and intra-subject and inter-subject CoV was 11.67% and 24.14%, respectively. Distance data extracted from the sensors during the reaching tasks were evaluated and then compared with the real movement distance. Regarding the accelerometer on the forearm sensor, the average of calculated movement distance was 34.14 ± 4.15 cm in the z-axis, and 33.54 ± 4.79 cm in the y-axis, where the measured distance in each direction was 34.0 cm and 33.5 cm, respectively. Data calculated from hand sensors were 36.78 ± 3.09 cm and 32.35 ± 4.64 cm), respectively. The intra-subject CoV ranged from 5.5% to 9.5%, whereas the inter-subject CoV ranged from 8.4% to 14.3%. The complete results are shown in Table 1. 

### 3.2. Workspace and RoM in Basic Upper Extremity Movements

All ten subjects were right-handed. For an orthogonal coordination, the axes were defined as follows: left-right direction for the x-axis, front-right direction for the y-axis, and up-down direction for the z-axis. For ARAT tasks, the size of the workspace for the right hand with reference to the sensor on the dorsum of the hand was 0.53 ± 0.11 m for the x-axis, 0.92 ± 0.08 m for the y-axis, and 0.89 ± 0.10 m for the z-axis. For the left side, the average workspace size was 0.62 × 0.80 × 0.86 m (in x, y, z-axis order). For pre-specified ADL tasks, the workspace for the dominant hand was 0.71 ± 0.22 m, 0.70 ± 0.17 m, and 0.86 ± 0.11 m (in x, y, z-axis order). The workspace of the non-dominant hand was significantly smaller, with an average size of 0.52 × 0.53 × 0.65 m (*p* = 0.001, 0.011, and 0.001 for the x-, y-, and z-axes, respectively). For the RoM in the major upper extremity joints, the angular ranges were similar between the right and left sides. The elbow flexion/extension and forearm supination/pronation showed the highest RoM in both ARAT and ADL for the dominant arm. The mean RoM values were 109.15 ± 18.82° and 105.23 ± 15.38° (elbow flexion/extension and forearm supination/pronation, respectively) for ARAT tasks, and 120.61 ± 23.64° and 128.09 ± 22.04° for ADL tasks. The RoM of the dominant side was significantly greater than on the non-dominant side for all joint directions except for the wrist dorsiflexion/volarflexion, which showed similar values (mean 113.70 ± 18.26° versus 110.08 ± 12.16°; right versus left, *p* = 0.526). All workspaces and RoM data during the ARAT and ADL tasks are shown in Table 2. 

### 3.3. Characteristics of Grasping/Pinching and Reaching

The upper extremity postures during grasping/pinching and reaching were analyzed as a subset analysis of the motion data extracted from grasping/pinching and reaching tasks in ARAT domains 1 and 3. Comparing grasping and pinching postures, the shoulder was more significantly abducted during pinching (19.39 ± 7.84°) compared to grasping (15.33 ± 6.91°, *p* = 0.040) and more extended during pinching (29.12 ± 12.33°) than grasping (22.99 ± 10.63°, *p* = 0.038). Elbow flexion/extension, forearm supination/pronation, and shoulder internal/external rotation did not significantly differ between the two postures. While reaching after grasping, the elbow was extended for an average of 87.87 ± 25.18° from the initial flexed posture, and pronated for an average of 36.65 ± 6.98° from the initial posture. The degrees of elbow extension and forearm pronation while reaching after pinching were similar (*p* = 0.849 and 0.294, respectively). The full results are shown in Table 3.

## 4. Discussion

The purpose of this study was to provide clinically relevant information regarding the workspace and major joint angle range while performing essential ADLs or important movements. By identifying these factors, it is possible to limit the extent of exoskeleton movements and, therefore, modify the design of the robot such that it can move within the designated workspace with a relatively simpler structure. In this study, we evaluated the RoM and workspace while conducting ARAT tasks, which is a common functional evaluation tool used in the clinics, because it is well known to significantly correlate with the patients’ functional status or recovery state [16,17,18]. ARAT consists of four domains: domain 1 and 3 tasks consist of the grasping and pinching of various sized objects and reaching afterwards. Domain 2 mainly involves moving items on a table focusing on the grip function, and domain four items are gross movement tasks that require lifting the arm to the head or face [19].

Validation of the IMU-based motion analysis system used in this study showed that the accuracy and reliability of the sensors themselves are very high regarding angles. However, in the form of wearable multi-sensor system, it is impossible to isolate a single joint movement, because all joints systemically move in three dimensions, including body trunk and contralateral upper extremity. Intra-subject covariance and inter-subject covariance were calculated for the forearm supination/pronation and elbow flexion/extension angles to evaluate the system reliability, and the range was deemed acceptable when considering that the reaching tasks were not completely identical. For the position data from the accelerometers, we compared the calculated data in the y and z directions using the estimated real movement distance. The calculated distance data were similar to the measured data, and the variability was considered acceptable. In addition, the calculated workspace and RoM during ARAT tasks were similar between the two extremities with no significant difference (Table 2). This may also support the reliability of the system-derived parameter values. Although it is difficult to state that the system provides a completely accurate measurement, it seems to provide consistent and meaningful data. 

The workspaces of the right and left hands were mostly similar, because ARAT repeats the same tasks with both hands alternatively. The slight difference between both sides is likely due to the difference in posture and orientation based on the limb dominance. During the ADL tasks, the workspace of the dominant hand, right hand for all subjects, was significantly larger than that of the non-dominant side by up to nearly 20 cm for all directions. In the view of stroke rehabilitation, most of the patients demonstrated hemiplegia of up to 80% or more [20], which means that their intact limb should be able to perform all normal functions. Patients with hemiplegia will use their intact hand as their dominant hand and, therefore, in certain occasions, the exoskeleton may only need to cover a smaller workspace than the dominant side. 

The RoMs of the major upper extremity joints during essential daily activities are presented in Table 2. The forearm supination/pronation and elbow flexion/extension showed the highest values for the dominant side. The RoM for the forearm supination/pronation was 128.09° and 108.00° on average for the right and left sides, respectively, during all ADL tasks. In a study using a reflective marker-based motion capture system, the whole RoM calculated by overlapping all 95% confidential interval range during various ADL tasks was approximately 92° [9]. Another study applied using an electromagnetic sensor system reported that the maximal supination angle from full pronation was 110° while glass drinking and 75° while combing their hair [8]. In a study by van Andel et al. [21], four selected ADL tasks were evaluated using an optic marker-based system, and their reported RoM for forearm supination/pronation was approximately 130°. Regarding elbow flexion/extension RoM, other studies also showed similar results. Aizawa et al. [8] reported a RoM of approximately 120° to 130° during various tasks, and Gates et al. [9] showed that the peak flexion angle of the elbow joint was 121° on average when drinking from a cup, which was the highest value among the evaluated tasks. Another study reported a RoM of around 140° from full extension [21]. Wrist dorsiflexion/volarflexion RoM was also similar with other studies, which ranged from 90° to 130°, whereas it was 113.70° and 110.08° for right and left side, respectively, in our study. It is important to ensure a sufficient RoM for elbow flexion/extension, forearm supination/pronation, and wrist dorsiflexion/volarflexion movements during rehabilitation, because such joint movements are essential for conducting ADL tasks, while recovery for distal joints are relatively slow and insufficient for a large portion of stroke patients [22,23,24].

The angular changes in the major joints when reaching after a grasping/pinching motion are evaluated because such actions are the fundamental movements for conducting any kind of tasks [25,26], and most of the activities are performed within the spatial range of these actions. Pinching was performed at a slightly but significantly more abducted and flexed posture of the shoulder joint, and showed a significant difference in fine tuning movements of the wrist joint. 

In contrast to a simple pure reaching movement, a reaching movement associated with a task may differ significantly regarding the arm postures, grasping position, and orientation [27,28]. The human motor system has high redundancy in terms of a multi-degree-of-freedom control system, and while task-relevant factors are specifically controlled, task-irrelevant variables are given relatively high variability [27]. In this study, the shoulder joint angles showed significantly different postures between grasping and pinching, which reflects different positions of the elbow joint while conducting a task. The wrist deviation and rotation angles also showed a significant difference, reflecting the difference in the fine motor posture and movements. Given the difference in posture, the main components of the reaching movement, elbow flexion/extension and forearm supination/pronation, did not differ significantly between the two types of tasks. This result may be applied to the swivel angle model suggested by Li et al. [27], where the shoulder joint angles can be simplified to a swivel angle regarding the orientation and posture, and the other distal joint angles account for essential reaching movements. In regular stroke rehabilitation, proximal muscle power recovery occurs in the early stage and more sufficiently compared to distal muscles [22,23,24] and, thus, it will be reasonable to motivate the patient to practice taking an appropriate posture for providing the right orientation of the upper extremity using the proximal muscles voluntarily, with the help of a gravity support system if applicable, whereas the individual robot joint actuation should focus on the essential distal joint movements such as elbow, forearm, and wrist movements.

Based on the current study dataset and the analysis, the workspace of the end-effector and its corresponding elbow or forearm position workspace, along with the essential elbow flexion/extension and forearm supination/pronation, may provide minimal structural requirements for the rehabilitation robot to maintain basic grasping, pinching, and reaching movements which are necessary to perform daily activities. This may be applied in both neurorehabilitation exoskeletons and assistive exoskeletons. These results may have helpful applications in virtual reality rehabilitation systems, especially in developing games or tasks which are clinically relevant.

This study has several limitations. To generalize the findings of the motion analysis, the number of subjects was relatively small. However, the statistical analyses provided minimal requirements regarding the validity and reliability and the data pattern for each subject was nearly identical especially during the structured movements (ARAT). Still, further studies with advanced protocol are necessary to verify and generalize the study results. In addition, gender and age factors could not be investigated sufficiently due to small number of subjects. Nevertheless, we have performed non-parametric tests to compare the major sensor-based parameters between men and women, and because the age range for this study was relatively in the young age, they were compared with additional dataset of the intact limb of the older people with hemiplegia. Most of the parameters did not show statistically significant difference according to the gender or age, except that older people tend to perform tasks within a smaller workspace during free ADL tasks and that women tend to abduct the shoulder more than men during pinching. IMU-based sensors basically have their own inevitable limitations, which include drift phenomenon in both the position and angular values, which may affect the values of the outcome measures [29]. In addition, a gimbal-lock phenomenon regarding the shoulder joint angles in particular may have occurred during the data measurements [8]. In this regard, the data may not be accurate in terms of the absolute values; however, because the data are sufficiently consistent, it seems that the general pattern of the data is reliable.

## 5. Conclusions

These study results provide the essential workspace and RoM of the major upper extremity joints during ARAT and ADL tasks in healthy subjects, which will serve as a basis in designing a practical and simple upper extremity exoskeleton robot. Further motion analyses on stroke patients are necessary to characterize upper extremity movements in neurological disorders and determine the key features in the stroke recovery process, which will be important in extracting the clinically relevant movement characteristics for designing new exoskeletons for neurorehabilitation purposes.

## Figures and Tables

**Table 1:** Coefficient of variation (CoV) for major movements ^a^.

Sensor Type	Axis	Average Change during Task (Across Subjects)	Intra-Subject CoV Average	Inter-Subject CoV	Estimated Real Distance *
Gyrosensor	Forearm supination/pronation	36.65 ± 6.98°	17.29%	19.05%	-
	Elbow flexion/extension	69.96 ± 16.89°	11.67%	24.14%	-
Accelerometer (forearm sensor)	z-axis distance (up/down)	34.14 ± 4.15 cm	6.18%	12.17%	34.0 cm
	y-axis distance (front/back)	33.54 ± 4.79 cm	7.16%	14.28%	33.5 cm
Accelerometer (hand sensor)	z-axis distance (up/down)	36.78 ± 3.09 cm	5.56%	8.41%	34.0 cm
	y-axis distance (front/back)	32.35 ± 4.64 cm	9.49%	14.33%	33.5 cm

^a^ Tasks performed by 10 normal subjects, six trials per reaching. * Estimated distance between initial object position and target position is approximately 33.5 cm for y-axis and 34.0 cm for z-axis. Note that this is the distance regarding common position of the forearm and hand sensors during the task and it varies by trials and subjects.

**Table 2:** Range of motion angle between right and left upper extremities during ARAT and ADL tasks.

	Axis	Right	Left	*p* ^a^
ARAT	x-axis (left-right, hand sensor)	0.53 ± 0.11 m	0.62 ± 0.07 m	0.082
	y-axis (front-back, hand sensor)	0.92 ± 0.08 m	0.80 ± 0.11 m	0.049 *
	z-axis (hand sensor)	0.89 ± 0.10 m	0.86 ± 0.08 m	0.224
	Shoulder abduction/adduction	50.16 ± 11.14°	55.34 ± 13.48°	0.249
	Shoulder flexion/extension	79.52 ± 19.34°	75.71 ± 21.56°	0.478
	Elbow flexion/extension	109.15 ± 18.82°	106.89 ± 12.83°	0.705
	Forearm supination/pronation	105.23 ± 15.38°	108.64 ± 12.64°	0.426
	Shoulder IR/ER	91.99 ± 20.98°	84.44 ± 44.75°	0.584
	Wrist dorsiflexion/volarflexion	82.90 ± 22.52°	81.26 ± 11.16°	0.833
ADL tasks	x-axis (left-right, hand sensor)	0.71 ± 0.22 m	0.52 ± 0.13 m	0.001 *
	y-axis (front-back, hand sensor)	0.70 ± 0.17 m	0.53 ± 0.15 m	0.011 *
	z-axis (hand sensor)	0.86 ± 0.11 m	0.65 ± 0.13 m	0.001 *
	Shoulder abduction/adduction	58.84 ± 14.53°	35.43 ± 10.09°	<0.001 *
	Shoulder flexion/extension	68.41 ± 17.56°	40.49 ± 18.54°	0.002 *
	Elbow flexion/extension	120.61 ± 23.64°	102.53 ± 19.51°	0.044 *
	Forearm supination/pronation	128.09 ± 22.04°	108.00 ± 16.23°	0.027 *
	Shoulder IR/ER	111.56 ± 31.88°	77.04 ± 21.28°	0.030 *
	Wrist dorsiflexion/volarflexion	113.70 ± 18.26°	110.08 ± 12.16°	0.526

^a^*p* value for paired *t* test between right and left side; * *p* value less than 0.05 considered statistically significant.

**Table 3:** Major joint angle position and change during grasping/pinching and reaching.

Axis	Grasping Initial Position	ROM during Reaching	*p*	Pinching Initial Position	ROM during Reaching	*p*	Grasp-Pinch *p* ^a^	Reaching Difference *p* ^b^
Shoulder abduction/adduction	15.33 ± 6.91° (abduction)	22.48 ± 19.81° (toward abduction)	0.006 *	19.39 ± 7.84° (abduction)	23.67 ± 13.35° (toward abduction)	<0.001 *	0.040 *	0.015 *
Shouler flexion/extension	22.99 ± 10.63° (extension)	47.80 ± 17.70° (toward flexion)	<0.001 *	29.12 ± 12.33° (extension)	41.83 ± 13.69° (toward flexion)	<0.001 *	0.038 *	0.948
Elbow flexion/extension	87.87 ± 25.18° (near fully flexed)	69.96 ± 16.89° (toward extension)	<0.001 *	84.82 ± 20.25° (near fully flexed)	67.91 ± 14.16° (toward extension)	<0.001 *	0.543	0.849
Forearm supination/pronation ^c^	34.37 ± 11.07° (supinated)	36.65 ± 6.98° (toward pronation)	<0.001 *	30.98 ± 13.71° (supinated)	36.02 ± 12.44° (toward pronation)	<0.001 *	0.181	0.294
Shoulder IR/ER	0.68 ± 23.56° (inward direction)	16.55 ± 23.02° (toward external rotation)	0.049 *	2.01 ± 13.74° (inward direction)	18.10 ± 13.02° (toward external rotation)	0.002 *	0.794	0.860
Wrist deviation	8.94 ± 12.12° (to thumb side)	−1.76 ± 10.21° (to finger side)	0.599	1.05 ± 8.19° (to thumb side)	4.81 ± 8.85° (to thumb side)	0.120	0.004 *	0.522
Wrist rotation	4.59 ± 7.35° (toward palm down)	7.12 ± 4.59° (toward palm up)	0.001 *	0.80 ± 5.25° (toward palm down)	4.75 ± 4.05° (toward palm up)	0.005 *	0.023 *	0.385
Wrist dorsiflexion/volarflexion	18.79 ± 16.35° (dorsiflexed)	6.79 ± 6.00° (toward volarflexion)	0.006 *	11.30 ± 13.90° (dorsiflexed)	7.28 ± 11.22° (toward volarflexion)	0.070	0.166	0.123

^a^ Comparison between grasping and pinching posture by paired *t* test; ^b^ Comparison between RoM change during reaching after grasping and pinching by paired *t* test; ^c^ Full pronation: 0°, full supination: 180°; * *p* value less than 0.05 considered statistically significant.

## Declaration

This manuscript is a revision of part of the first author’s (Nam HS) Ph. D. thesis from Seoul National University.
